# Oral immunotherapy for peanut allergy: a systematic review and meta-analysis

**DOI:** 10.1097/MS9.0000000000004974

**Published:** 2026-06-09

**Authors:** Muhammad Waaiz, Waniya Badar Khan, Rimsha Adnan, Shiza Abid, Muhammad Saad Khurshid, Jamal Nasir Karim, Saira Jahangir Khan, Eman Javed, Syeda Laiba Fahim, Verkha Kumari, Muzainah Tabassum, Qurat Ul Ain Muhammad, Pratik Bhattarai

**Affiliations:** a Department of Medicine, Dow University of Health Sciences, Karachi, Pakistan; b Department of Medicine, Ayub Medical College, Abbottabad, Pakistan; c Department of Medicine, Shaheed Mohtarma Benazir Bhutto Medical College Lyari, Karachi, Pakistan; d Department of Medicine, Liaquat National Hospital and Medical College, Karachi, Pakistan; e Department of Medicine, Jinnah Sindh Medical University, Karachi, Pakistan; f Department of Medicine, Rawalpindi Medical University, Rawalpindi, Pakistan; g Department of Medicine, Manipal College of Medical Sciences, Pokhara, Nepal

**Keywords:** anaphylaxis, desensitization, epinephrine, oral immunotherapy, peanut allergy, placebo

## Abstract

**Background and objectives:**

Food allergy, particularly peanut allergy, is a significant and growing health concern, especially in high-income countries. Affecting 2% of children and 1% of adults, peanut allergy is a chronic condition that severely impacts quality of life. The standard treatment remains allergen avoidance, though oral immunotherapy (OIT) has emerged as a potential strategy for desensitization. This systematic review and meta-analysis aims to evaluate the efficacy and safety of peanut oral immunotherapy (POIT) based on randomized controlled trials (RCTs).

**Methods:**

A systematic review and meta-analyses were conducted following PRISMA guidelines. Eligible studies included double-blind RCTs evaluating POIT versus placebo or avoidance. Databases such as PubMed, Google Scholar, Cochrane-Controlled Register of Trials, and ClinicalTrials.gov were searched. Risk of bias was assessed using the Cochrane Risk of Bias tool (ROB2), and statistical analysis was performed using the RevMan software.

**Results:**

A total of 20 studies with 2161 participants (median age: 8.6 years) were included. POIT demonstrated a significant increase in desensitization rates (RR = 7.25, 95% CI: 2.66–19.79, *P* = 0.0001). However, POIT was also associated with increased risks of anaphylaxis (RR = 2.27, 95% CI: 1.48–3.47, *P* = 0.0002) and epinephrine use (RR = 2.05, 95% CI: 1.35–3.12, *P* = 0.0008). Adverse effects such as gastrointestinal symptoms, respiratory events, and skin abnormalities were more frequent in the POIT group, leading to a higher treatment discontinuation rate (RR = 2.50, 95% CI: 1.20–5.21, *P* = 0.01).

**Conclusion:**

POIT is effective in inducing desensitization in peanut-allergic individuals but carries significant risks, including an increased likelihood of anaphylaxis and adverse events. These findings reinforce previous meta-analyses and highlight the need for individualized risk-benefit assessments in clinical practice. Further research is required to optimize treatment protocols and improve patient safety.

## Introduction

This study was conducted and reported in accordance with the TITAN (Transparent Reporting of Time-series Forecasting and Trend Analyses) guidelines to ensure transparency, reproducibility, and methodological rigor^[^1^]^. Food allergy is an escalating global health concern with the greatest prevalence in high-income countries like the USA, where it is estimated to affect up to 8% of children and 2–3% of adults^[^2,3^]^. Food allergy is a hypersensitivity response caused by Immunoglobulin E (IgE) antibody to an allergen present in food^[^4,5^]^. It usually starts during childhood and may persist to adulthood, greatly impacting the quality of the individual’s life^[^3,4^]^. The most common foods inflicting anaphylaxis are cow’s milk, hen’s egg, peanut, tree nut, wheat, soy, fish, and shellfish; however, in recent times, peanut allergy has emerged as the most prevalent and significant^[^3,5^]^.

Peanut allergy, being the most prevalent nutrient allergy, has become a chief cause of food allergic reactions, anaphylaxis, and death^[^2^]^. It now affects 2% of children and 1% of adults in high-income countries^[^2,3^]^. Peanut allergy is a chronic condition that profoundly impacts the lifestyle of the affected individuals^[^6^]^. The standard of medical care is allergen avoidance and acute intervention; however, the extensive occurrence of peanuts in food products has increased the risk of anaphylaxis, which is a critical and potentially life-threatening emergency^[^2^]^.

Oral immunotherapy (OIT) has emerged as an effective treatment for desensitizing children with food allergies^[^6^]^. It involves the administration of progressively larger doses of allergen to the patient under medical surveillance to build tolerance^[^3^]^. This process begins with low doses, which are systematically increased until a stable maintenance dose is established^[^3^]^. The Food and Drug Administration in 2020 approved a peanut allergen powder, Palforzia (previously DNFP), a novel OIT product for treating peanut allergy in children^[^7,8^]^. Peanut oral immunotherapy (POIT) aims to desensitize patients, thereby reducing the risk of allergic reactions (12% per year) and anaphylaxis (7% per year)^[^2^]^.

In recent years, various trials and studies have been conducted on POIT. A Cochrane review evaluating the effectiveness and safety of OIT for peanut allergy, including randomized controlled trials (RCTs), was published in 2019^[^2^]^. Since then, additional RCTs have been performed. Therefore, the aim of our study is to systematically review, analyze, and reassess the clinical impact of OIT on the treatment of peanut allergy.

## Methods

This systematic review and meta-analysis was conducted based on the Preferred Reporting Items for Systematic Reviews and Meta-Analyses (PRISMA) statement^[^9^]^, Grading of Recommendations, Assessment, Development and Evaluation (GRADE), and Cochrane guidelines. This study is registered in an international prospective registry (registration number blinded for review). The work has been reported in line with AMSTAR (Assessing the methodological quality of systematic reviews) Guidelines.

### Eligibility criteria

Double-blind RCTs studying the effects of POIT on individuals with peanut allergies were included. Literature reviews, case reports, and non-randomized trials were excluded. We placed no restrictions on the age of diagnosis of peanut allergy but kept a median population age of 3–15 years. Studies enrolling participants with a previously diagnosed peanut allergy and a current allergic status through skin prick tests were included. Since we investigated the efficacy and safety of POIT, the intervention had to be the consumption of peanut protein in the form of a pill or powder in one group and a placebo in the other group.

We prioritized outcomes that were essential to the patients, particularly focusing on peanut allergy occurrences outside of the clinical provocation test setting. A surrogate measure of treatment efficacy is the percentage of patients who passed the supervised and appraised oral food challenge (clinic provocation test) with the grading criteria defined by each individual study. We hypothesized that POIT would reduce food allergic reactions, so we included more direct measures such as peanut-induced anaphylaxis, peanut-induced allergic reactions, and epinephrine use. We categorized allergic reactions based on the organ systems involved, their severity, and whether they led to treatment cessation.

Our outcomes to investigate were (1) desensitization to peanut protein, (2) incidence of anaphylaxis along with the (3) use of epinephrine, (4) skin and subcutaneous tissue disorders (contact urticaria, flush, generalized pruritus, dermatitis), (5) digestive events (abdominal pain, vomiting, nausea), (6) serious adverse events, including those that may have caused treatment discontinuation, (7) lower respiratory tract events (coughing, wheezing, shortness of breath/dyspnea), and (8) upper respiratory tract events (rhinitis, sneezing, rhino conjunctivitis, nasal congestion). All adverse events were a result of exposure to the treatment.

Moreover, due to language restrictions, we only included studies published in English.

### Data sources and literature search

**Figure 1.**
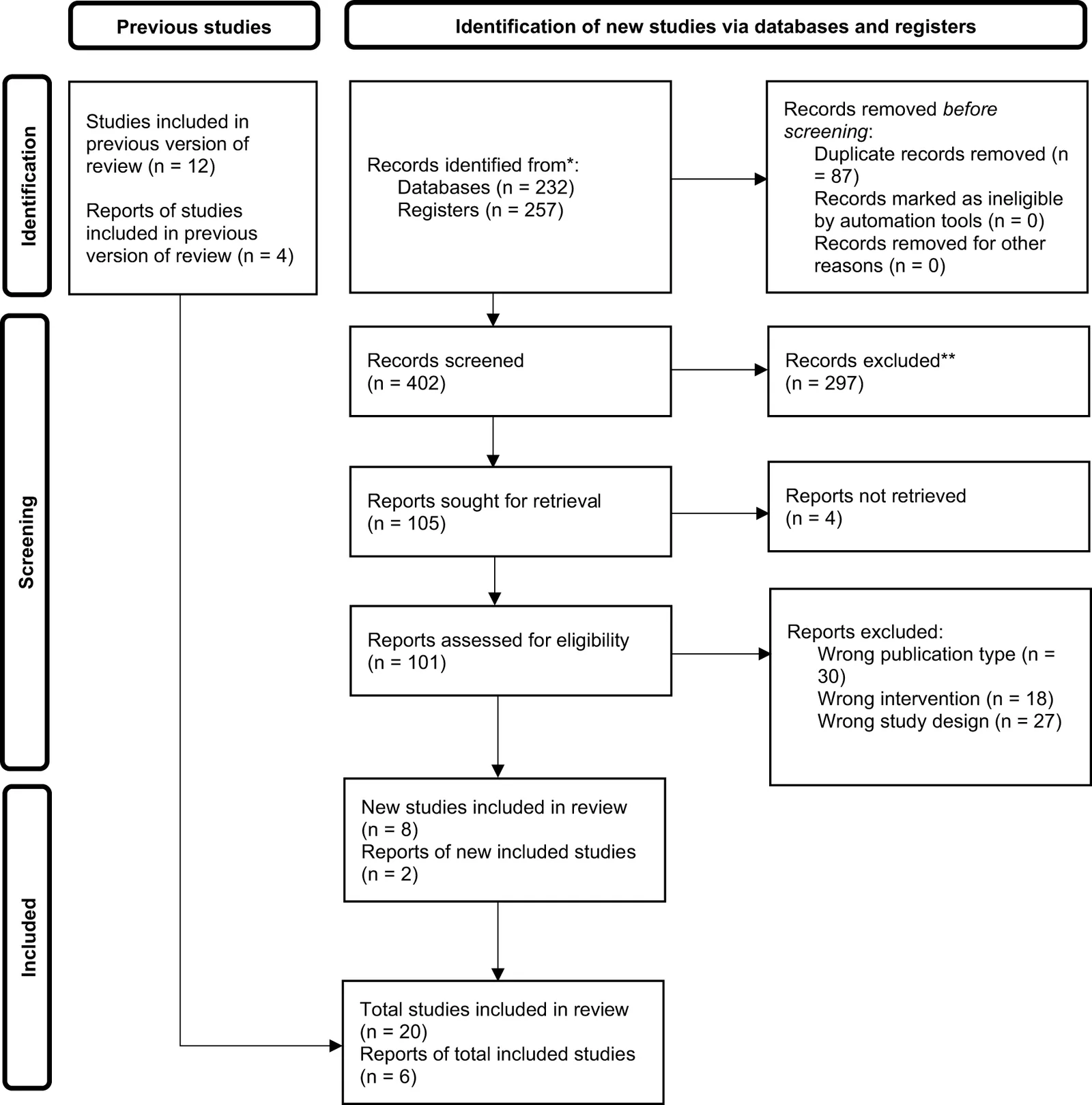
PRISMA flowchart of the database literature search.

We searched PubMed (MEDLINE), Cochrane-Controlled Register of Trials, and ClinicalTrials.gov for published RCTs^[^2^]^. Specific and relevant keywords like “oral immunotherapy” and “peanut allergy” were used in the search strategy. Furthermore, the references mentioned in the retrieved studies were assessed to identify other studies that met our inclusion criteria (Supplemental Digital Content Table S1, available at: http://links.lww.com/MS9/B177). Our database searches yielded a total of 3906 studies. After removal of 367 duplicates, we screened 3539 publications and excluded 3455; retrieved 84 in full-text and removed 64 to include 20 total studies and reports of included studies (Fig. 1).

HIGHLIGHTS

- 20 studies, 2161 participants (median age 8.6 years; 37% female, 63% male).
- Compared proprietary OIT (6 trials) and non-proprietary OIT (14 trials) to placebo (13 trials), avoidance (6 trials), or sublingual immunotherapy (1 trial).
- Effectiveness: 1061 of 1557 participants achieved desensitization (RR = 7.25, 95% CI 2.66–19.79, *P* = 0.0001).○Shorter treatment (≤12 months) showed higher success (RR = 11.59) compared to >12 months (RR = 4.02).
- Side effects:○Anaphylaxis: RR = 2.27 (*P* = 0.0002).○Epinephrine use: RR = 2.05 (*P* = 0.0008).○Gastrointestinal symptoms: abdominal pain (RR = 2.11), vomiting (RR = 1.96), and nausea (RR = 2.15).○Respiratory symptoms: cough/wheeze (RR = 1.42) and upper respiratory events (RR = 1.47).○Skin reactions: RR = 1.49.
- Discontinuation: 136 of 1178 OIT participants versus 16 of 504 controls (RR = 2.50, *P* = 0.01).

We undertook our screening through the web application Rayyan^[^10^]^. The screening process began by selecting the studies based on their abstract. Following this, those studies that passed the initial screening underwent a secondary screening process with their full texts retrieved and examined. Duplicates were identified by Rayyan and removed.

### Bias assessment

We assessed the quality of the included RCTs using the Cochrane Risk of Bias tool for randomized controlled trials (ROB2)^[^11^]^. The tool assesses the quality of randomized trials by examining five key domains: bias arising from the randomization process, bias due to deviations from intended interventions, bias due to missing outcome data, bias in measurement of the outcome, and bias in the selection of the reported result. Each domain is evaluated using signaling questions, and judgments are made as “low risk,” “some concerns,” or “high risk” of bias (Supplemental Digital Content Figure S1a and b, available at: http://links.lww.com/MS9/B169). We used the GRADE approach to evaluate the certainty or quality of the studies included. The GRADE system classifies evidence based on confidence in its accuracy. High-certainty evidence means we are sure the true effect is similar or close to the estimate. Moderate-certainty evidence suggests the true effect is probably close to the estimate but could vary. Low-certainty evidence shows limited confidence, so the true effect might differ significantly from the estimate. Very low-certainty evidence shows minimal confidence, suggesting the true effect is antithetical to the estimate. We employed GRADEpro GDT to create a summary of finding tables (Supplemental Digital Content Table S2, available at: http://links.lww.com/MS9/B178). Publication bias was determined by statistically examining funnel plots by Egger’s test (Supplemental Digital Content Figure S2a–k, available at: http://links.lww.com/MS9/B170).

### Data synthesis and statistical analysis

We analyzed outcomes data using the intention-to-treat (ITT) approach. If multiple reports were published from similar data, we considered the report with the latest results available. For dichotomous outcomes, we combined the data using the risk ratio (RR) and incidence RR. Data were analyzed using the RevMan software. Effect sizes and 95% confidence interval (CI) for the intervention were calculated using a random-effects model across studies. Forest plots were generated to evaluate the effect of POIT on each of the outcomes. To check the validity of the results, leave-one-out sensitivity analysis was conducted to assess if any single study disproportionately influenced the results and resulted in an increase in heterogeneity. We performed subgroup analyses for the main outcomes based on the control group (placebo or avoidance) accordingly. The subgroups included were the median age of participants, proprietary or non-proprietary OIT, starting dose, target dose and duration of OIT. Heterogeneity was assessed using the Cochrane *Q* statistic; *P* < 0.1 indicates significant heterogeneity. Heterogeneity across all trials was also evaluated by the *I*^2^ test, and the scale was set as a value <25% = low risk, 25–75% = moderate risk, and >75% = high risk. A *P*-value of <0.05 was considered significant in all cases.

## Results

### Study characteristics and demographic characteristics of participants

This systematic review and meta-analysis includes a total of 20 studies (Table 1). Included studies enrolled 2161 participants, with an average median age of participants across trials of 8.6 years. The studies consist of 37.3% female and 62.7% male participants undergoing POIT (6 trials with proprietary products^[^12–17^]^, and 14 with non-proprietary products^[^18–31^]^), versus no OIT (13 placebo trials^[^13–18,21–23,25,26,29,30^]^, 6 avoidance trials^[^12,20,24,27,28,31^]^, and 1 sublingual immunotherapy trial^[^19^]^). POIT was given in all the trials, and the average starting dose was 147 mg daily, with an average target dose of 1895 mg.

**Table 1:** Baseline characteristics of included trials and participants.

	Study	Country	Type of study	OIT group	Proprietary	No OIT group	Starting dose (mg)	Target dose (mg)	Treatment duration (months)	No. of participants	Mean age, years	Women, *n* (%)
**1**	Varshney *et al* (2011)^[^18^]^	USA	RCT	OIT	No	Placebo	0.1	4000	11.3–16.3	28	5.75	10 (36)
**2**	STOP II (2014)^[^12^]^	UK	RCT	OIT	Yes	Avoidance	2	800	5.9	99	12.4	29 (29)
**3**	PPOIT (2015)^[^13^]^	Australia	RCT	PPOIT	Yes	Placebo	0.1	2000	18	62	5.95	25 (40)
**4**	Narisety *et al* (2015)^[^19^]^	USA	RCT	OIT	No	Sublingual immunotherapy	0.1	2000	–	21	11.1	10 (48)
**5**	Kukkonen *et al* (2016)^[^20^]^	Finland	RCT	OIT	No	Avoidance	0.1	800	8	60	8.5	25 (41)
**6**	ARC001 (2017)^[^14^]^	USA	RCT	OIT	Yes	Placebo	0.5	300	5	55	7.5	19 (35)
**7**	PMIT (2017)^[^21^]^	USA	RCT	OIT	No	Placebo	–	4000	36–60	10	5.4	3 (30)
**8**	PnOIT3 (2017)^[^22^]^	USA	RCT	OIT	No	Placebo	–	4000	60	16	5	10 (62.5)
**9**	Nagakura *et al* (2017)^[^23^]^	Japan	RCT	OIT	No	Placebo	795	795	24	33	8.5	4 (18.2)
**10**	PNOIT (2018)^[^24^]^	USA	RCT	OIT	No	Avoidance	–	4000	>36	30	–	12 (40)
**11**	Blumchen *et al* (2018)^[^25^]^	Germany	RCT	OIT	No	Placebo	0.5	125–250	15.2	62	6.8	24 (39)
**12**	PALISADE (ARC003) (2018)^[^15^]^	North America and Europe	RCT	OIT	Yes	Placebo	0.5	300	12	551	11.3	236 (43)
**13**	PITA (2018)^[^26^]^	France	RCT	GIDOIT	No	Placebo	2	400	5.5	30	14.75	8 (27)
**14**	TAKE-AWAY (2018)^[^27^]^	Norway	RCT	OIT	No	Avoidance	1	5000	12.9	77	9.5	33 (43)
**15**	Nagakura *et al* (2018)^[^28^]^	Japan	Non-RCT	OIT	No	Avoidance	8	133	12	34	8.6	9 (26.47)
**16**	POISED (2019)^[^29^]^	USA	RCT	OIT	No	Placebo	300	300/4000	24	120	11	19 (32)
**17**	ARTEMIS (ARC010) (2020)^[^16^]^	Europe	RCT	OIT	Yes	Placebo	0.5	1000	9	175	7.5	80 (45.7)
**18**	RAMSES (ARC007) (2021)^[^17^]^	North America	RCT	OIT	Yes	Placebo	0.5	300	6	505	9	186 (36.6)
**19**	IMPACT (2022)^[^30^]^	USA	RCT	OIT	No	Placebo	0.1	5000	25.6	146	3.2	47 (32)
**20**	BOPI-1 (2022)^[^31^]^	UK	RCT	OIT	No	Avoidance	>1400	>4400	12	47	12.5	20 (42.6)

OIT, oral immunotherapy; PPOIT, probiotic peanut oral immunotherapy; USA: United States of America; UK: United Kingdom; RCT, randomized controlled trial.

### Risk of bias summary

Eight studies^[^12,14,18,23,24,27–29^]^ clearly state that the participants were randomly assigned to receive active therapy or controlled intervention to reduce bias. Three studies^[^18,28,29^]^ state that the investigators, participants, and caregivers were also blinded until the study was completed. None of the studies deviated from the intended intervention. Moreover, none of the studies had any missing outcomes or showed any evidence of bias that could result in the obscuring of outcomes. The overall risk for bias is low (Supplemental Digital Content Figure S1a and b, available at: http://links.lww.com/MS9/B169).

### Primary outcome

#### Desensitisation

The primary outcome of our analysis was desensitization of the stimulus post-treatment. Nineteen studies (*n* = 1656) reported desensitization^[^12–16,18–31^]^, out of which 13 reported outcomes with a significant effect size^[^12–18,20,25,26,29–31^]^. The RR is as follows: [RR = 7.25 (95% CI, 2.66, 19.79)] and a *P* value of 0.0001 showing that the results are statistically significant and proving the effect is due to administration of OIT. A total of 1557 subjects were randomized, of which 1061 were successfully desensitized post-treatment. The heterogeneity for this outcome is considerably high (*I*^2^ = 93%).

**Figure 2.**
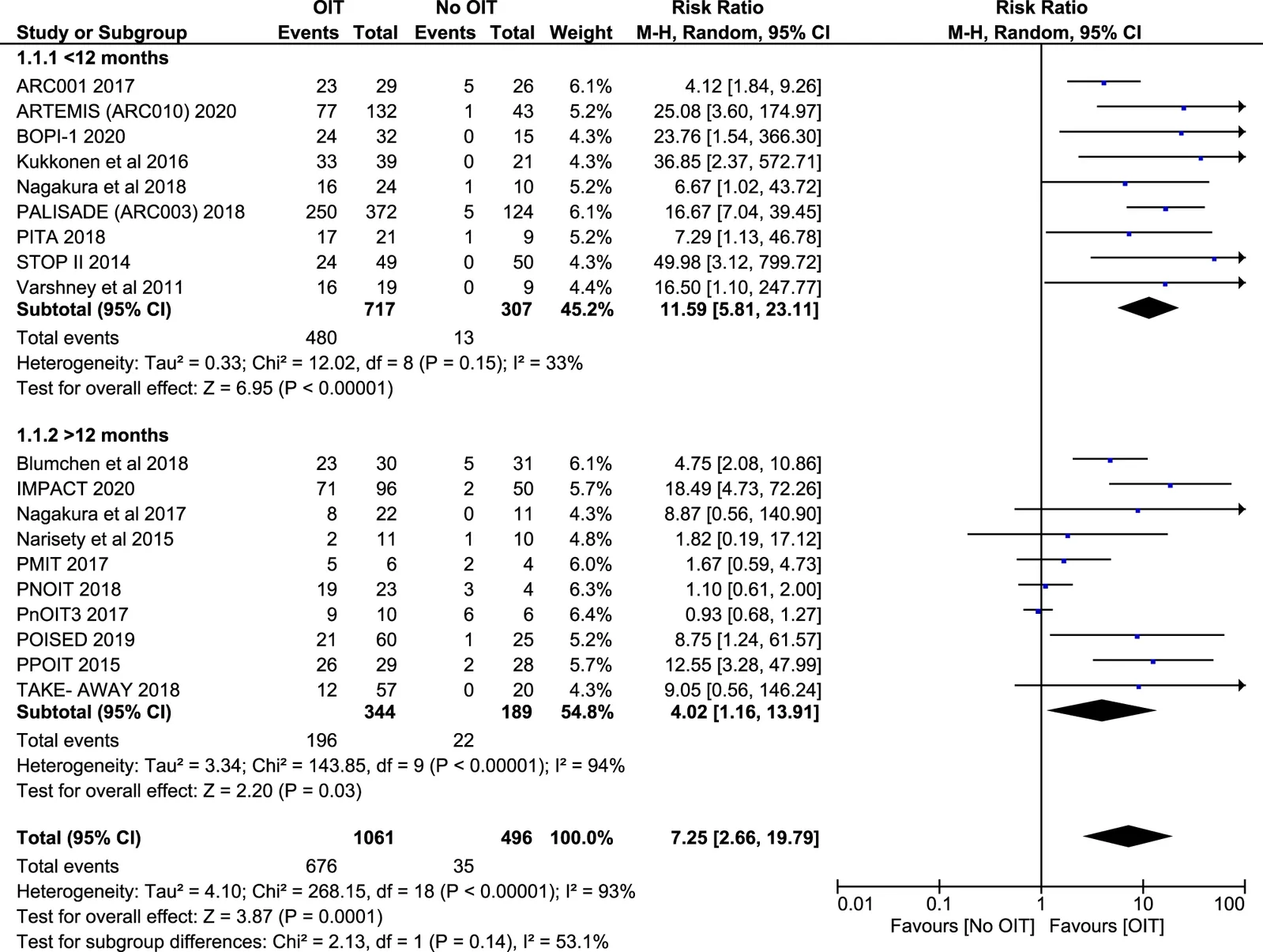
Forest plot showing desensitization to peanut allergy after OIT versus placebo for peanut allergy.

We performed a subgroup analysis for treatment duration, with the subgroups being “more than 12 months” and “equal to or less than 12 months.” We found that an increase in treatment duration above 12 months would decrease the number of participants successfully undergoing desensitization. The results are as follows: [RR = 4.02 (95% CI, 1.16, 13.91)], *P* = 0.03, *I*^2^ = 94%. For studies where treatment duration was equal to or less than 12 months, [RR = 11.59 (95% CI, 5.81, 23.11), *P* < 0.00001, *I*^2^ = 33%]. The final heterogeneity and overall effect for subgroup differences is *P* = 0.0001, *I*^2^ = 53.1%. No other subgroup differences were found for proprietary or non-proprietary OIT, target dose, starting dose, age groups, and country of origin. However, some of the studies contrast large RR, exhibiting that there were adverse side effects of OIT (Fig. 2).

### Secondary outcomes

#### Anaphylaxis

**Figure 3.**
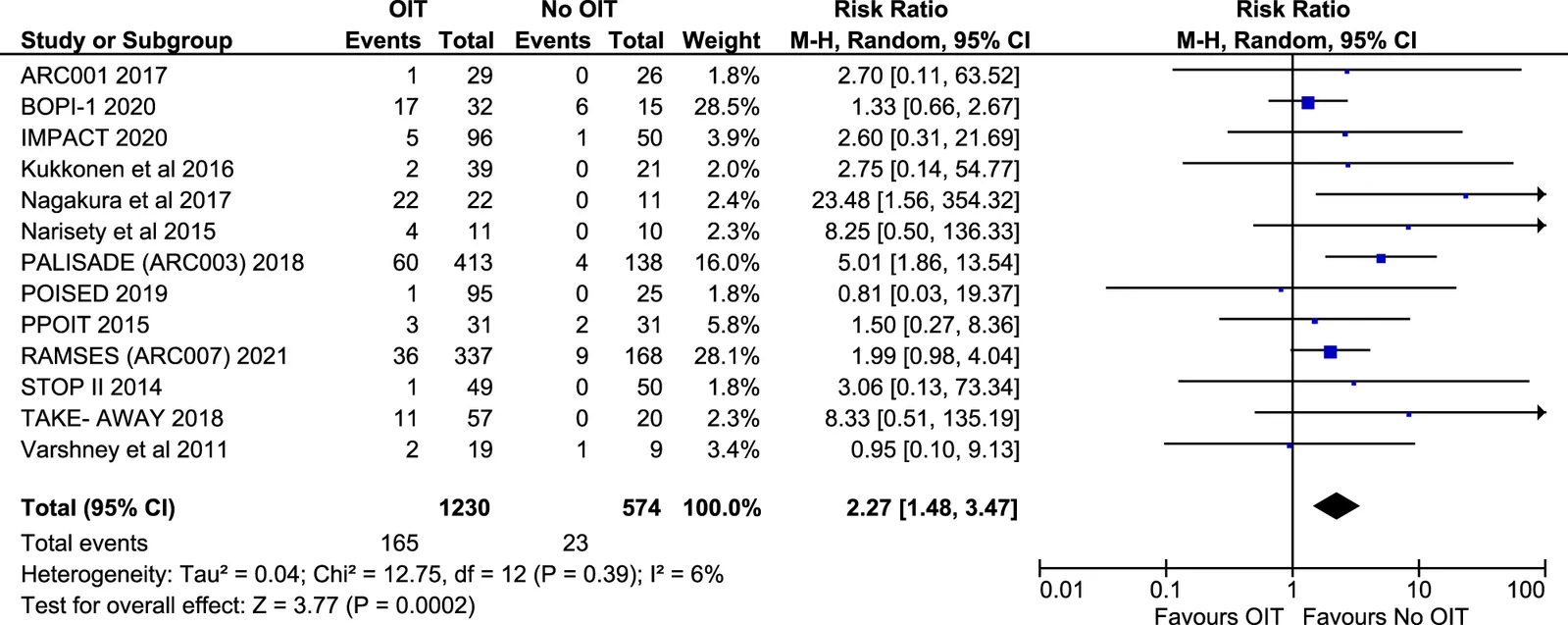
Forest plot showing the incidence of anaphylaxis to peanut allergen after OIT versus placebo for peanut allergy.

Thirteen studies (*n* = 1804) reported anaphylaxis, which was significantly inclined toward OIT^[^12–15,17–20,23,27,29–31^]^ affecting 165 participants. Participants receiving OIT were more likely to experience anaphylaxis [RR = 2.27 (95% CI, 1.48, 3.47), *P* = 0.0002, *I*^2^ = 6%]. We found no subgroup effects for treatment duration, proprietary or non-proprietary OIT, target dose, starting dose, age groups, and country of origin (Fig. 3).

#### Epinephrine use

**Figure 4.**
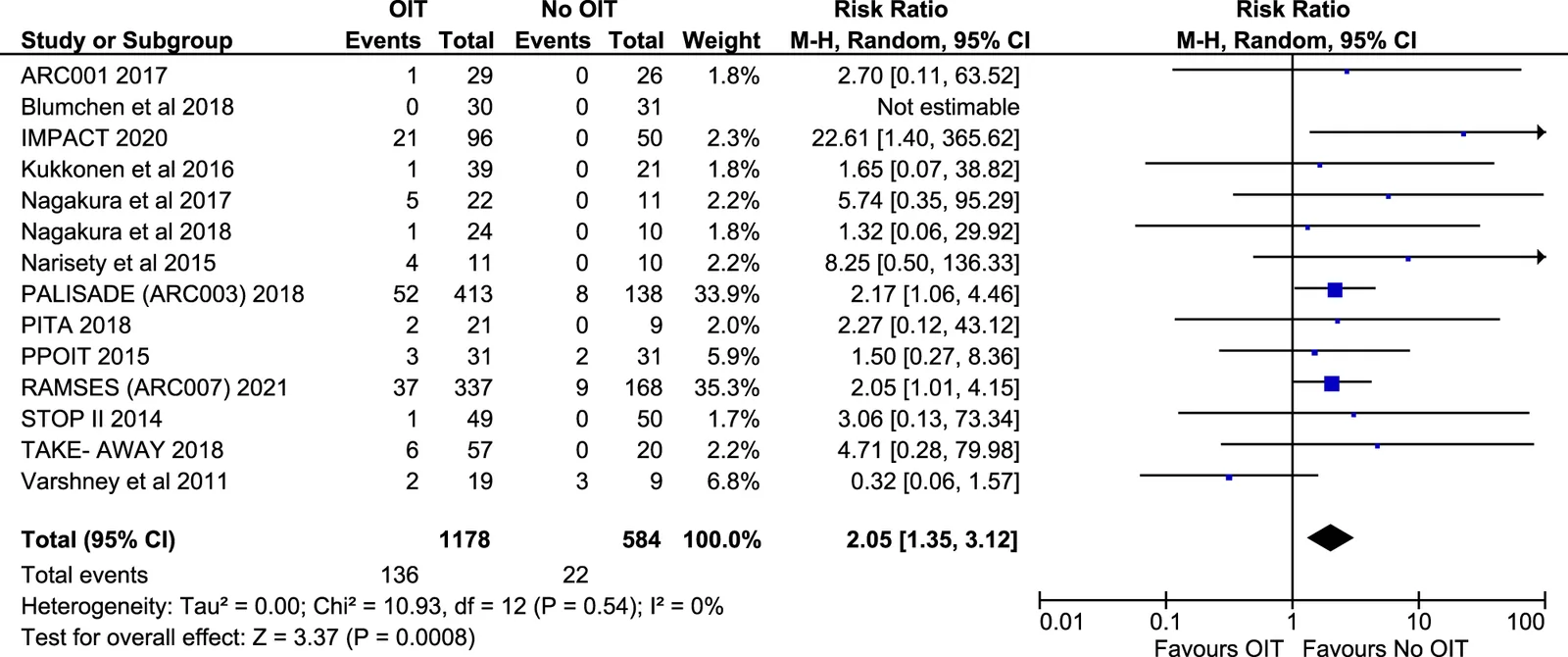
Forest plot showing the incidence of epinephrine use to peanut allergen after OIT versus placebo for peanut allergy.

Fourteen studies (*n* = 1762) reported the use of epinephrine by 158 participants^[^12–15,17–20,23,25–28,30^]^. OIT increased the use of epinephrine [RR = 2.05 (95% CI, 1.35, 3.12), *P* = 0.0008, *I*^2^ = 0%] (Fig. 4).

#### Digestive events (nausea, vomiting, and abdominal pain)

Abdominal pain was reported by 13 studies (*n* = 1750)^[^13–20,22–25,30^]^, and only 655 events were given an account of [RR = 2.11 (95% CI, 1.79, 2.48), *P* = 0.00001, *I*^2^ = 69%] (Supplemental Digital Content Figure S3a, available at: http://links.lww.com/MS9/B171).

Vomiting was reported by 9 studies (*n* = 1495)^[^13–17,20–22,25^]^, and 451 events were reported [RR = 1.96 (95% CI, 1.58, 2.43), *P* < 0.00001] (Supplemental Digital Content Figure S3b, available at: http://links.lww.com/MS9/B171).

Nausea was reported by 6 studies (*n* = 1407)^[^14–17,20,25^]^, and 399 events were reported [RR = 2.15 (95% CI, 1.69, 2.72), *P* < 0.00001] (Supplemental Digital Content Figure S3c, available at: http://links.lww.com/MS9/B171).

The incidence of these symptoms was observed to be very low in all studies. This greatly implies that the evidence for such symptoms was insufficient, and hence the results are insignificant and require more studies.

#### Adverse events causing discontinuation

Eleven studies (*n* = 1595) included the record of discontinuation by subjects^[^12,15,17–19,24–27,30,31^]^. 136 subjects from 1178 discontinued OIT due to its adverse effects, while 16 out of 504 left the placebo, no OIT group [RR = 2.50 (95% CI, 1.20, 5.21), *P* = 0.01, *I*^2^ = 41%] (Supplemental Digital Content Figure S4, available at: http://links.lww.com/MS9/B172).

#### Lower respiratory tract (coughing and wheezing)

Twelve studies (*n* = 1675) reported effects on the lower respiratory tract with symptoms such as coughing and wheezing^[^13–17,21–25,28,30^]^ with OIT being the major contributing factor [RR = 1.42 (95% CI, 1.03, 1.92), *P* = 0.03, *I*^2^ = 43%]. 353 participants out of 1153 exhibited such symptoms in the OIT group compared to 102 out of 1522 in the placebo, no OIT group. On the other hand, there is less evidence reported for other symptoms of the lower respiratory tract, such as dyspnea/shortness of breath^[^13,15–17,20,30^]^ (Supplemental Digital Content Figure S5, available at: http://links.lww.com/MS9/B173; Supplemental Digital Content Figure S6, available at: http://links.lww.com/MS9/B174).

#### Upper respiratory tract

Ten studies (*n* = 1609) recorded upper respiratory effects^[^13–18,21–25,28,30^]^. Comparatively, the OIT group did show a greater number of events (289 out of 1103) compared to the No OIT group (83 out of 506) [RR = 1.47 (95% CI, 1.19, 1.81), *P* = 0.0003] (Supplemental Digital Content Figure S7, available at: http://links.lww.com/MS9/B175).

#### Skin abnormalities

Thirteen studies (*n* = 1773) recorded skin abnormalities^[^13–18,20–22,24,25,27,30^]^, and almost half of the OIT group reported events of symptoms (e.g., rash). 505 out of 1222 in the OIT group, while 130 out of 551 participants had such symptoms in the placebo, no OIT group [RR = 1.49 (95% CI, 1.08, 2.04), *P* = 0.01, *I*^2^ = 65%] (Supplemental Digital Content Figure S8, available at: http://links.lww.com/MS9/B176).

## Discussion

This systematic review and meta-analysis of over 2000 peanut allergic patients from 20 RCTs provides valuable insights on the safety and efficacy of POIT compared with allergen avoidance or placebo. This study included 1438 participants in the OIT group and 638 participants in the control group.

Our findings revealed that POIT significantly induced our primary outcome of desensitization. Despite achieving successful desensitization, our pooled analysis demonstrated that POIT elevated the risk of anaphylaxis, adverse events, and epinephrine use. Additionally, digestive symptoms (including abdominal pain, nausea, and vomiting), as well as upper and lower respiratory tract symptoms, were more frequent. POIT is also linked to skin abnormalities and some treatment discontinuation; however, it is not associated with an increased risk of developing severe adverse events.

Our results are consistent with those reported in a previous meta-analysis published by Chu *et al*^[^2^]^ with 8 RCTs and data on 1041 total participants. Our study demonstrated an absolute risk of developing anaphylaxis of 9.1% (5.93–13.90), which is close to the risk reported by Chu *et al* of 8.4% (4.74–14.94). Similarly, the risk associated with the use of epinephrine documented by Chu *et al* was 8.2% (4.7–14.2), which strongly aligns with the risk observed in our study of 7.7% (5.08–11.75).

The meta-analysis by de Silva *et al*^[^32^]^ demonstrated a significant increase in tolerance of peanuts during OIT [RR = 9.9 (95% CI, 4.5, 21.4, high certainty)]; however, they did not find an increase in the risk of developing anaphylaxis due to OIT. Moreover, due to scarcity, they did not include respiratory, skin and gastrointestinal disorders in their analysis.

A recent clinical trial has demonstrated that the early immunological alterations that contribute to peanut desensitization include dynamic changes in Treg cells and potential perturbations in monocytes, NK cells, and B cells. Furthermore, single-cell RNA sequencing analyses have indicated alterations in gene expression in naïve and memory gdTreg populations during initial stages of OIT in children. An increase in the expression of genes associated with immune regulation and tolerance, such as CD69, ISG20, CTLA4, and TGFB, was observed in memory gdTregs^[^33^]^.

Participants in our analysis had a median age of 8.6 years. Data on adult POIT is scarce, and the results obtained have not been encouraging. The POISED study^[^27^]^, which was included in our meta-analysis, enrolled 120 patients aged 7–55 years (median age 11 years) receiving peanut OIT and included a subgroup of 22 adults. Even though this RCT was not designed to compare differences between adults and children, the results demonstrated that there were no differences in safety, efficacy, and risk of adverse events between these two groups. However, the dropout rate reported in adults was 32% and was notably higher compared to the 9% that was observed in children. Similarly, the AR101 study^[^14^]^ enrolled 20 participants who received active treatment and 13 participants received a placebo, aged 18–55 years.

The results demonstrated that the difference between the desensitization rate in the active treatment group (41.5%) and the placebo group (14.3%) was not statistically significant. Furthermore, the dropout rate reported in the adult cohort was higher compared to the rate seen in children (4–17 years).

A retrospective study conducted by Rigbi *et al*^[^34^]^ analyzed data of 96 adults who received OIT for IgE-mediated food allergy, including peanuts, and compared it with the results for children (4 to <11 years) and adolescents (≥11 to 17 years). Their findings highlighted that the increased risk for treatment failure in adults is not solely attributed to the adult age, but rather to more severe allergy profiles in this population. Adults exhibited significantly higher skin prick test results compared to the younger individuals, and this has been associated with a more challenging OIT course^[^35^]^.

Furthermore, our study aligns with findings from other forms of peanut immunotherapy. A recent meta-analysis conducted by Banatwala *et al*^[^36^]^ enrolled six trials on epicutaneous immunotherapy and demonstrated a significant benefit in achieving desensitization [RR: 2.13 (95% CI, 1.72, 2.64), *P* < 0.01, *I*^2^ = 0%] when administered 250 µg of epicutaneous immunotherapy (EPIT). However, they found no significant association between EPIT and adverse events such as skin reactions, respiratory symptoms, anaphylaxis, or serious adverse events.

The meta-analysis conducted by Xiong *et al*^[^37^]^ also showed significant benefit in terms of desensitization by peanut EPIT (RR 2.34, 95% CI 1.69–3.23, *I*^2^ = 0%) however they reported that the incidence of anaphylactic reactions in the EPIT group, categorized by organ systems (including skin and upper and lower respiratory tract), and the use of rescue medications was similar to the control group. In contrast, our findings revealed that OIT significantly increases the incidence of these outcomes (high-certainty evidence).

The previous analysis employed a comprehensive search across multiple databases and included a wide range of studies on OIT for peanut allergy^[^29^]^. It adhered to strict methodological guidelines. For instance, RCTs with ITT analyses were prioritized, minimizing bias and increasing the reliability of findings on safety and efficacy^[^38^]^. Use of the GRADE approach to evaluate evidence quality further enhanced transparency and confidence in the results^[^29,39^]^. However, the meta-analysis had limitations. For example, the sample sizes of some included studies were relatively small compared to larger clinical trials. This constraint could limit the generalizability of the findings and lead to overestimation or underestimation of treatment effects. Smaller trials may not fully represent the diversity of individuals with peanut allergies, potentially skewing efficacy and safety evaluations^[^40^]^. Additionally, there is a lack of long-term follow-up studies assessing the persistence of desensitization or sustained unresponsiveness after the cessation of OIT^[^40,41^]^.

While this analysis demonstrates considerable strengths, such as the broad review of desensitization outcomes, certain limitations remain. High heterogeneity among studies (*I*^2^ = 93%) undermines the reliability of pooled results, even when subgroup analyses are performed. Moreover, incomplete data reporting for specific outcomes, such as the RR for anaphylaxis, further weakens the conclusions. Addressing these shortcomings in future research – particularly by reducing heterogeneity – will be critical. Despite these challenges, the strengths of this meta-analysis lie in its thorough evaluation across multiple POIT studies. Nevertheless, large-scale trials are necessary to assess long-term benefits and refine treatment protocols.

Given the demonstrated conflict between increased desensitization and higher rates of anaphylaxis among other adverse events, implementation of POIT should involve a structured and individualized risk-benefit assessment. Candidates for POIT may be prioritized among children with persistent peanut allergies who are at a high risk for accidental exposure and have access to specialized allergy care. Pre-treatment evaluations should assess baseline allergy severity using skin prick tests and peanut-specific IgE levels, while also considering history of prior reactions and comorbid conditions such as uncontrolled asthma that could be further exacerbated by peanut allergen. Taking these factors into account can help clinicians streamline treatment protocols and anticipate potential complications. Patients and caregivers should be counseled extensively regarding the increased likelihood of mild to moderate reactions, the potential need for epinephrine use, and appropriate emergency management during therapy. To ensure safety, POIT should be administered in centers built for emergency response, with regular follow-ups to monitor tolerance, adjust dosing, and detect adverse events early. Additionally, incorporating patient-reported outcomes and preferences can further optimize selection and minimize adverse events, supporting shared decision-making between clinicians and families. Care should be taken in adult patients, where limited data and higher treatment discontinuation rates may influence the risk-benefit profile.

## Conclusion

When compared to allergen avoidance, POIT significantly increases desensitization, but it also increases the risk of anaphylaxis, adverse events, and treatment discontinuation, according to this meta-analysis. POIT did not increase severe adverse events, although most reactions were mild to moderate. These results, which are in line with earlier analyses and backed by larger pooled data, reinforce the evidence for POIT’s effectiveness while highlighting its safety issues. Short follow-up, limited adult data, and high study heterogeneity continue to be major drawbacks. Large-scale, standardized trials in the future are required to maximize safety and efficacy and elucidate long-term results.

## Data Availability

All data generated is available within the manuscript or the supplementary file.

## Research registration unique identifying number (UIN)

This study is registered in PROSPERO as CRD420250543579.
